# A Two-Step Immunomagnetic Microbead-Based Method for the Isolation of Human Primary Skin Telocytes/CD34+ Stromal Cells

**DOI:** 10.3390/ijms21165877

**Published:** 2020-08-16

**Authors:** Eloisa Romano, Irene Rosa, Bianca Saveria Fioretto, Elena Lucattelli, Marco Innocenti, Lidia Ibba-Manneschi, Marco Matucci-Cerinic, Mirko Manetti

**Affiliations:** 1 Department of Experimental and Clinical Medicine, Division of Rheumatology, University of Florence, 50134 Florence, Italy; eloisaromano@libero.it (E.R.); biancafioretto@icloud.com (B.S.F.); marco.matuccicerinic@unifi.it (M.M.-C.); 2 Department of Experimental and Clinical Medicine, Section of Anatomy and Histology, University of Florence, 50134 Florence, Italy; irene.rosa@unifi.it (I.R.); lidia.ibba@unifi.it (L.I.-M.); 3 Plastic and Reconstructive Microsurgery, Careggi University Hospital, 50134 Florence, Italy; elena.lucattelli@gmail.com (E.L.); marco.innocenti@unifi.it (M.I.)

**Keywords:** telocytes, CD34+ stromal cells, skin, cell isolation, immunomagnetic separation

## Abstract

Telocytes (TCs), commonly referred to as TCs/CD34+ stromal cells, are a peculiar type of interstitial cells with distinctive morphologic traits that are supposed to exert several biological functions, including tissue homeostasis regulation, cell-to-cell signaling, immune surveillance, and reparative/regenerative effects. At present, the majority of studies investigating these cells are mainly descriptive and focus only on their morphology, with a consequent paucity of functional data. To gain relevant insight into the possible functions of TCs, in vitro analyses are clearly required, but currently, the protocols for TC isolation are only at the early stages and not fully standardized. In the present in vitro study, we describe a novel methodology for the purification of human primary skin TCs through a two-step immunomagnetic microbead-based cell separation (i.e., negative selection for CD31 followed by positive selection for CD34) capable of discriminating these cells from other connective tissue-resident cells on the basis of their different immunophenotypic features. Our experiments clearly demonstrated that the proposed method allows a selective purification of cells exhibiting the peculiar TC morphology. Isolated TCs displayed very long cytoplasmic extensions with a moniliform silhouette (telopodes) and presented an immunophenotypic profile (CD31−/CD34+/PDGFRα+/vimentin+) that unequivocally differentiates them from endothelial cells (CD31+/CD34+/PDGFRα−/vimentin+) and fibroblasts (CD31−/CD34−/PDGFRα+/vimentin+). This novel methodology for the isolation of TCs lays the groundwork for further research aimed at elucidating their functional properties and possible translational applications, especially in the field of regenerative medicine.

## 1. Introduction

Telocytes (TCs) are a long-neglected peculiar type of stromal cells that were firstly described by Popescu and Faussone-Pellegrini in 2010 [1] and that have been subsequently identified, over the last decade, in both perivascular and interstitial compartments of a variety of organs in humans and other vertebrates [2,3,4,5,6,7,8,9,10,11,12]. Based on their unique ultrastructural morphology revealed by transmission electron microscopy (TEM), TCs have been originally defined as “stromal cells with telopodes”, as they present a small nucleated cell body giving rise to extremely long cytoplasmic prolongations (telopodes) with a characteristic moniliform aspect due to the alternation of very slender segments (podomers) and bead-like dilated portions (podoms) accommodating mitochondria, endoplasmic reticulum cisternae, and caveolae [1,2,5,10]. TCs are able to release a variety of extracellular microvesicles containing bioactive molecules and, through their telopodes, they form complex three-dimensional networks within the stromal compartment of different organs, either by interconnecting to each other or establishing multiple cell–cell contacts with other cell types, such as immune cells, muscle fibers, blood vessels, and epithelial cells [5,10,13,14]. Given their peculiar morphology, spatial distribution, and secretory abilities, TCs have been proposed to exert multiple functional roles in their resident tissues, including structural support and tissue organization during development and post-natal homeostasis, juxtacrine and paracrine cell-to-cell signaling, immune surveillance, and reparative/regenerative effects by supporting local stem cell niche maintenance and differentiation [2,4,5,15,16,17,18,19,20,21,22,23]. 

Although the lack of TC-specific antigenic markers still makes TEM the gold-standard technique to identify TCs within tissues, increasing evidence demonstrates that TCs from virtually every human organ consistently express the cell surface glycoprotein CD34, making them detectable also by immunohistochemical analyses [5,6,7,10,11]. Indeed, according to substantial literature, TCs are also commonly referred to as TCs/CD34+ stromal cells [6,7,20]. However, as CD34 expression is not exclusive to TCs, when immunolocalizing these cells by light microscopy, it is preferable to use a combination of multiple markers. In particular, since CD34 is strongly expressed also by vascular endothelial cells (ECs), CD34/CD31 double immunostaining has been proven to be extremely useful to unequivocally distinguish between CD34+/CD31− TCs and CD34+/CD31+ ECs of blood vessels, especially considering that the latter, when captured in tissue sections, may often exhibit an elongated profile resembling that of TCs [7,8,9,24,25]. Another marker commonly used to identify TCs by immunohistochemistry is platelet-derived growth factor receptor (PDGFR)α that has been demonstrated to be coexpressed with CD34 in TCs from different organs [5,8,9,10,11,25,26,27].

Given the extensive distribution of TC interstitial networks in different organs and the numerous putative roles attributed to these stromal cells, increasing literature has hypothesized that their phenotypic and functional alterations may be implicated in different pathologic conditions [28,29,30,31]. Indeed, recent studies have reported a reduction in TCs and severe structural changes in the TC stromal network in a variety of disorders, including heart failure and conditions characterized by chronic tissue inflammation and/or fibrotic remodeling, such as psoriasis, primary Sjögren’s syndrome, Crohn’s disease, ulcerative colitis, systemic sclerosis, and liver fibrosis [24,28,32,33,34,35,36,37,38].

Despite the growing belief that TCs may exert important roles in both physiologic and pathologic conditions, at present, the majority of studies investigating these cells are mainly descriptive and focus only on their morphology, with a consequent paucity of functional data. To gain relevant insight into the possible functions of TCs, in vitro analyses are clearly required, but currently, the protocols for TC isolation are only at early stages and not fully standardized [39,40,41,42,43,44,45,46]. The present study was, therefore, undertaken to develop, for the first time, a methodology for the selective in vitro isolation of human primary TCs/CD34+ stromal cells consisting in a two-step immunomagnetic microbead-based separation of these cells from two other stromal cell populations, namely ECs and fibroblasts. 

## 2. Results

### 2.1. TC Purification from Healthy Human Skin and In Vitro Assessment of Cell Morphology 

**Figure 1:**
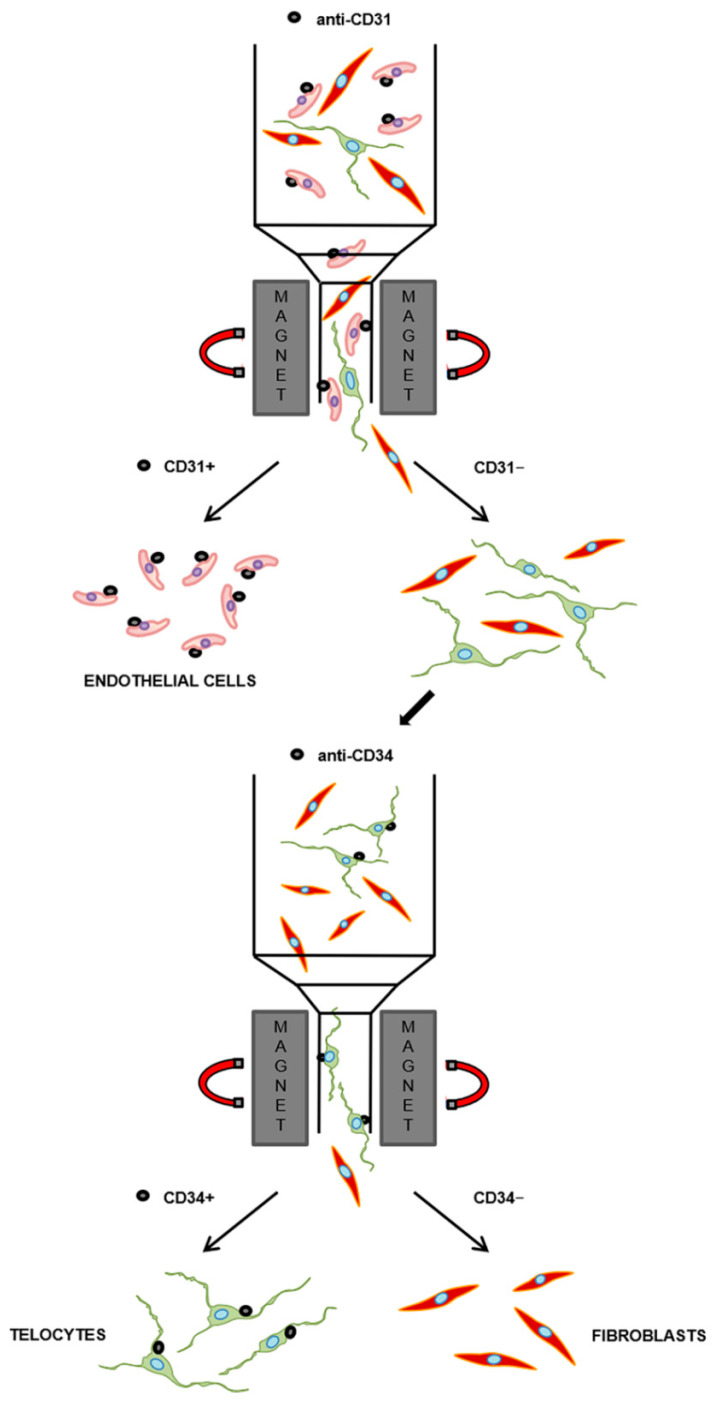
Schematic representation of the two-step immunomagnetic microbead-based cell separation protocol. Total dermal cells obtained from the processing of healthy human skin samples were subjected to magnetic-activated cell sorting separation. The first step was carried out in order to isolate CD31+ cells (i.e., putative endothelial cells), while the second step, performed on the pool of the remaining CD31− cells, allowed the further separation of CD31−/CD34+ cells (i.e., putative telocytes) from CD31−/CD34− cells (i.e., putative fibroblasts).

In order to establish a TC purification protocol required to allow reliable future in vitro investigations, we designed a two-step immunomagnetic cell separation method based on our current knowledge of the immunophenotypic features of these cells [6,7,8,9]. Since the most widely used TC marker, namely CD34, is also expressed by ECs, the first step of the protocol consisted of a microbead-based cell selection for the pan-endothelial cell surface marker CD31, also known as platelet/endothelial cell adhesion molecule-1, to separate CD31+ cells (i.e., putative ECs) from CD31− cells (Figure 1). The second step, performed on the pool of the remaining CD31− cells, allowed their further selection on the basis of the expression of the CD34 cell surface antigen to purify CD31−/CD34+ cells (i.e., putative TCs) from CD31−/CD34− cells (i.e., putative fibroblasts) (Figure 1). Given the ease of collection, to set up our protocol for the isolation of primary TCs/CD34+ stromal cells, we employed specimens of healthy human skin, a tissue in which these peculiar cells have been reported to be widely distributed throughout the dermal layer, where they intimately surround vessels and skin appendages, and establish numerous intercellular communications with neighboring cells, including fibroblasts [34,47,48,49,50]. 

**Figure 2:**
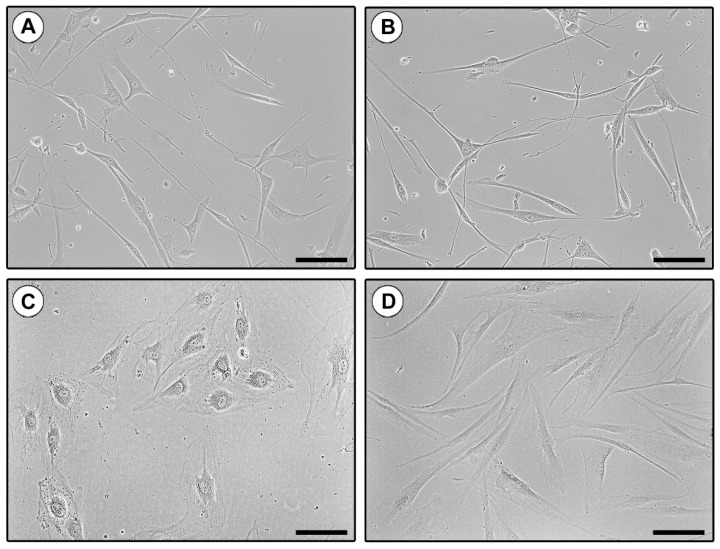
Morphologic assessment of in vitro cultured CD31−/CD34+, CD31+, and CD31−/CD34− immunomagnetically selected cells by phase-contrast microscopy. (**A**,**B**) Representative phase-contrast microphotographs of CD31−/CD34+ purified cells showing the distinctive telocyte morphology, with a small nucleated cell body giving rise to extremely long cytoplasmic prolongations with a moniliform appearance (i.e., telopodes). (**C**) Representative phase-contrast microphotograph of CD31+ isolated cells exhibiting the characteristic polygonal cobblestone-like morphology of ECs. (**D**) Representative phase-contrast microphotograph of CD31−/CD34− isolated cells presenting the typical spindle shape of fibroblasts. Original magnification: ×20 (**A**–**D**). Scale bar: 100 µm (**A**–**D**).

The identity of the three different immunomagnetically purified cell populations (CD31+ putative ECs, CD31−/CD34+ putative TCs, and CD31−/CD34− putative fibroblasts) was firstly evaluated by placing them into culture and subsequently, assessing their specific cell morphology using both phase-contrast microscopy and wheat germ agglutinin (WGA) fluorescent staining. As shown in Figure 2A,B, CD31−/CD34+ sorted cells presented the distinctive TC morphology, with a small nucleated cell body, from which extremely long and moniliform cytoplasmic prolongations (telopodes) abruptly arise. Conversely, CD31+ cells exhibited the characteristic polygonal cobblestone-like morphology of ECs (Figure 2C), while CD31−/CD34− cells showed the typical spindle shape of fibroblasts, with shorter and thicker non-moniliform cytoplasmic prolongations (Figure 2D).

**Figure 3:**
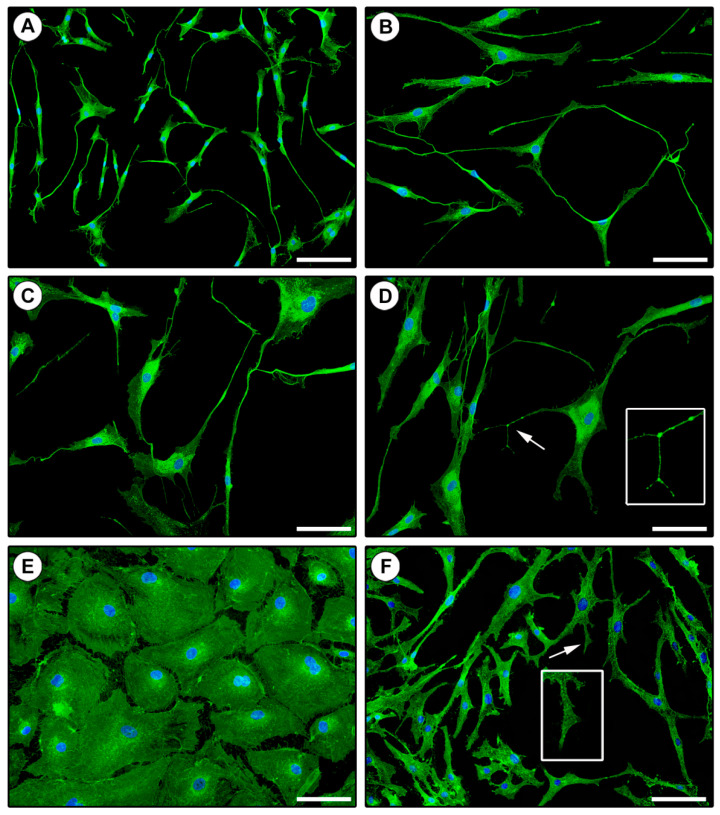
Morphologic assessment of in vitro cultured CD31−/CD34+, CD31+, and CD31−/CD34− immunomagnetically selected cells by staining of the cell membrane with fluorescent wheat germ agglutinin. (**A**–**D**) Representative fluorescent microscopic images of CD31−/CD34+ isolated cells exhibiting the distinctive telocyte morphology, with a small cell body and long cytoplasmic prolongations (i.e., telopodes) presenting a moniliform aspect conferred by the alternation of slender segments (podomers) and bead-like dilated portions (podoms). Telocytes are heterogeneous in the shape of their cell body that gives rise to a variable number of telopodes. (**D**) A higher magnification view of the telopode pointed by the arrow is shown in the inset. (**E**) Representative fluorescent image of CD31+ isolated cells with the characteristic polygonal cobblestone-like morphology of endothelial cells. (**F**) Representative fluorescent image of CD31−/CD34− isolated cells presenting the typical spindle shape of fibroblasts, with shorter and thicker non-moniliform cytoplasmic prolongations. The fibroblast prolongation pointed by the arrow is shown at higher magnification in the inset. Nuclei are counterstained in blue with 4′,6-diamidino-2-phenylindole (DAPI). Original magnification: ×10 (**A**), ×20 (**B**–**F**). Scale bar: 200 µm (**A**), 100 µm (**B**–**F**).

To further characterize the morphologic features of the three isolated and in vitro cultured cell populations, we next performed WGA fluorescent staining. Indeed, WGA conjugates have the ability to bind the cellular glycocalyx and, therefore, are routinely used to label the plasma membrane highlighting the cellular shape. As displayed in Figure 3, cell morphologies detected by WGA staining were comparable to those observed by phase-contrast microscopy. In particular, CD31−/CD34+ purified cells exhibited very long cytoplasmic prolongations with a clear moniliform aspect conferred by the alternation of slender segments and bead-like dilated portions, which are distinctive features of TCs (Figure 3A–D). Similarly, CD31+ and CD31−/CD34− isolated cells stained with WGA presented the typical morphologic characteristics of ECs (Figure 3E) and fibroblasts (Figure 3F), respectively.

### 2.2. Immunophenotypic Characterization of In Vitro Cultured CD31−/CD34+, CD31+, and CD31−/CD34− Immunomagnetically Selected Cells

**Figure 4:**
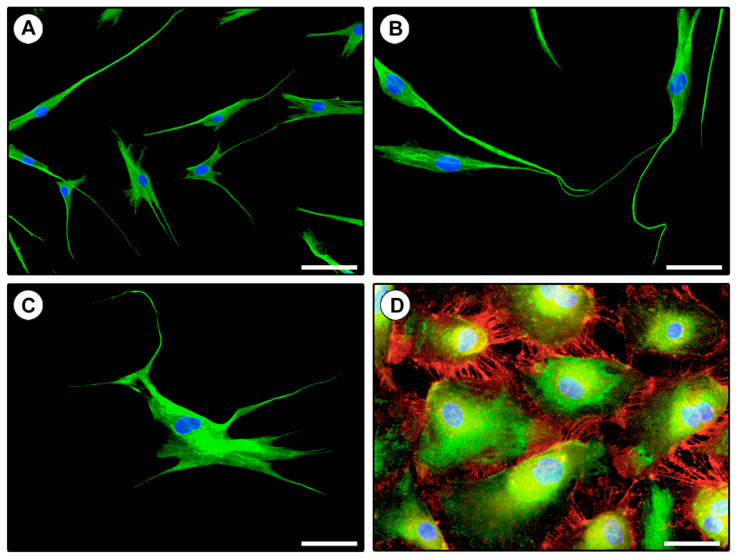
Verification of the immunophenotypic profile of in vitro cultured CD31−/CD34+ and CD31+ immunomagnetically selected cells by CD31/CD34 double immunofluorescence. (**A**–**C**) Representative fluorescent microscopic images of CD31−/CD34+ immunomagnetically selected cells double immunostained for CD34 (green) and CD31 (red). The cells express only CD34 and are morphologically identifiable as telocytes with typical telopodes. The cell body of telocytes varies in shape, being oval, triangular, or spindle-shaped, and gives rise to a variable number of telopodes. (**B**) Note the presence of telocytes establishing intercellular contacts with their telopodes. (**D**) Representative fluorescent image of CD31+ immunomagnetically selected cells double immunostained for CD34 (green) and CD31 (red). All cells coexpress CD31 and CD34 and display the typical polygonal cobblestone-like morphology of endothelial cells. Note the distinctive localization of the CD31 antigen at regions of intercellular contacts. Nuclei are counterstained in blue with 4′,6-diamidino-2-phenylindole (DAPI). Original magnification: ×20 (**A**), ×40 (**B**–**D**). Scale bar: 100 µm (**A**), 50 µm (**B**–**D**).

In order to confirm the effectiveness of our immunomagnetic separation protocol, the three different isolated cell populations were further subjected to a verification of their immunophenotypic profile through CD31/CD34 double immunofluorescence staining. As shown in Figure 4A–C, cells negatively selected for CD31 during the first step of purification and subsequently subjected to CD34 positive selection were effectively immunolabeled only with the anti-CD34 antibody and displayed the typical TC morphology. Cells negatively selected for both CD31 and CD34 (i.e., fibroblasts) resulted completely unlabeled. Finally, CD31+ selected cells were found to coexpress CD31 and CD34, thus, confirming their EC identity (Figure 4D).

The purity of immunomagnetically isolated CD31−/CD34+ TCs was ≥90%, as assessed by determining the percentage of cells immunolabeled for CD34, but not CD31, and displaying the typical TC morphology with respect to the total number of cells identified by DAPI-labeled nuclei.

**Figure 5:**
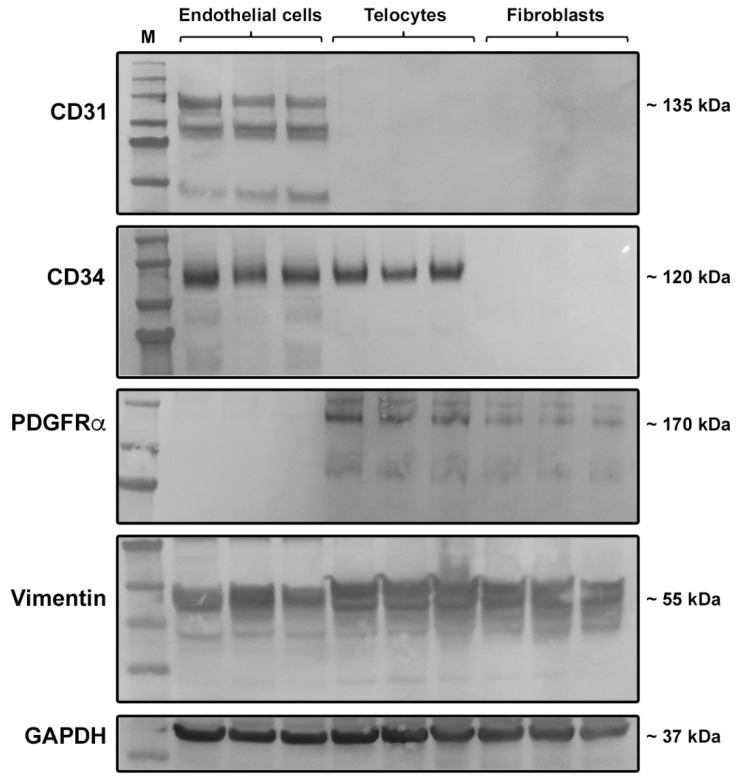
Western blotting analysis of CD31, CD34, platelet-derived growth factor receptor (PDGFR)α, and vimentin protein expression in CD31+ (endothelial cells), CD31−/CD34+ (telocytes), and CD31−/CD34− (fibroblasts) immunomagnetically selected cells. Protein lysates of the three different immunomagnetically purified cell populations were assayed for the expression of CD31, CD34, PDGFRα, and vimentin. Representative immunoblots are shown. Glyceraldehyde 3-phosphate dehydrogenase (GAPDH) was measured as a loading control. Molecular weight values (kDa) are indicated on the right. M—molecular weight marker.

The immunophenotype of the three different immunomagnetically purified cell populations was further assessed through the analysis of the protein expression of CD31, CD34, PDGFRα, and vimentin by Western blotting. According to the immunofluorescence findings, cells positively selected for CD31 (i.e., ECs) were found to express both CD31 and CD34 proteins, while cells negatively selected for CD31 during the first step of immunomagnetic purification and then, subjected to positive selection for CD34 (i.e., TCs) expressed CD34 but not CD31 (Figure 5). Western blotting analysis also confirmed that in CD31−/CD34− immunomagnetically selected cells (i.e., fibroblasts), neither of the two antigens were expressed (Figure 5). As far as PDGFRα expression is concerned, it was detected in both TCs and fibroblasts, but not in ECs (Figure 5). Of note, in agreement with substantial literature [5,8,9,10,11,25,26,27,51], the coexpression of CD34 and PDGFRα further confirmed that the CD31−/CD34+ immunomagnetically selected cell population consisted of TCs (Figure 5). We also evaluated the protein expression of vimentin, a marker that is often used to stain TCs [4,43,52]. As shown in Figure 5, vimentin protein was found in all the three isolated cell populations, which is consistent with the evidence that this marker is widely expressed by all cells of mesenchymal origin, including TCs, fibroblasts as well as ECs, which indeed are the only mesenchyme-derived epithelial cell type [53,54].

**Figure 6:**
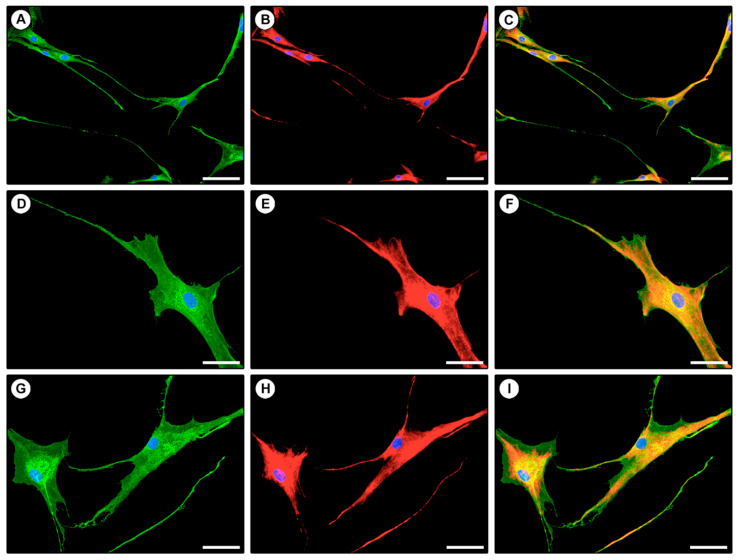
Coexpression of CD34 and PDGFRα in isolated telocytes assessed by double immunofluorescence. (**A**–**I**) Representative fluorescent microscopic images of purified telocytes double immunostained for CD34 (green) and PDGFRα (red). Single green (**A**,**D**,**G**), single red (**B**,**E**,**H**) and merged (**C**,**F**,**I**) images are shown. Nuclei are counterstained in blue with 4′,6-diamidino-2-phenylindole (DAPI). Original magnification: ×20 (**A**–**C**), ×40 (**D**–**I**). Scale bar: 100 µm (**A**–**C**), 50 µm (**D**–**I**).

Finally, we performed double immunofluorescence staining to confirm the coexpression of CD34 and PDGFRα in isolated TCs displaying typical telopodes (Figure 6).

## 3. Discussion

The present in vitro study describes a novel methodology for the selective purification of human primary TCs/CD34+ stromal cells through a two-step immunomagnetic microbead-based cell separation capable of discriminating these cells from other connective tissue-resident cells (i.e., ECs and fibroblasts) on the basis of their different immunophenotypic profile. Indeed, we clearly demonstrate that TCs can be easily isolated from human healthy skin and that, when cultured, these cells exhibit their peculiar morphology, with very long telopodes. Moreover, we also provide evidence that magnetically isolated TCs are CD31−/CD34+/PDGFRα+/vimentin+, immunophenotypic features that are consistent with the current literature [5,6,7,8,9,10,11,24,25,26,27,51], and clearly differentiate these stromal cells from ECs (CD31+/CD34+/PDGFRα−/vimentin+) and fibroblasts (CD31−/CD34−/PDGFRα+/vimentin+). Of note, TCs from some organs have also been shown to express CD117/c-kit [5,7]. However, here, CD117/c-kit was not included in the TC molecular profiling because in a previous work, we have clearly demonstrated that dermal TCs/CD34+ stromal cells are negative for this marker [34].

Isolation and culture expansion of TCs are of great interest in the perspective of allowing future reliable morphofunctional in vitro investigations to gain fundamental insight into the functions of these peculiar type of interstitial cells. In fact, the majority of studies that have so far investigated TCs in physiologic and pathologic tissues are mainly descriptive and focused on the ultrastructural, morphologic, and immunophenotypic characterization of these cells within their resident tissues, leaving much of their proposed functions still a matter of debate [22,29,55,56]. In this regard, the ability of TCs to form complex interstitial networks and communicate with neighboring cells by establishing multiple intercellular contacts or releasing extracellular vesicles containing bioactive molecules, together with the evidence that such TC networks are severely impaired in a variety of pathologies, led to proposing that these cells may exert a wide spectrum of functions important for the maintenance of tissue homeostasis [2,4,5,10,28,29,57,58]. For instance, owing to their strategic position in relation to tissue-resident stem cell niches in various organs and satellite cells in the skeletal muscle, it has been postulated that TCs may play a pivotal role in stem cell survival, proliferation, differentiation, maturation, and guidance, eventually stimulating tissue regenerative processes [5,19,52,59,60,61,62]. Furthermore, TCs are usually found to intimately surround microvessels with their telopodes and may express proangiogenic growth factors, such as vascular endothelial growth factor, and several microRNAs with angiogenic functions, suggesting that they may influence EC functions contributing to vascular homeostasis and neovascularization processes [41,63,64]. TCs might also have an active role in immunomodulation/immune surveillance, as they have been described to establish cell-to-cell contacts with immune cells and to be damaged and reduced in different pathologies characterized by acute or chronic inflammation such as salpingitis, psoriasis, and inflammatory bowel diseases [3,5,24,35,36,65,66]. Ultrastructural alterations and loss of TCs have also been reported during fibrotic tissue remodeling, which led to attributing them a possible role in the regulation of neighboring fibroblast functionality [28,34,37,38]. In addition, TCs seem to possess electrophysiologic properties, as reported in myometrium, myocardium, and the enteric neuromuscular compartment, where they have been proposed to contribute to the spreading of the slow waves generated by the pacemaker interstitial cells of Cajal [2,5,26,44,58,67,68]. In light of the above, it is clear that the possibility to use purified and in vitro expanded TCs in coculture with other cell populations, including stem cells, ECs, immune cells, and fibroblasts, may represent an extremely useful tool to definitively unravel the effective functional roles and implications of these stromal cells in different pathologic conditions. Of note, over the past few years, several attempts have been made to isolate TCs from various organs such as the uterus [39,44,46], lung [45], and heart [41,42,43]. Nevertheless, it is important to consider that all these studies discerned TCs only on the basis of their different adhesion time on the plate, placing into culture the cells remaining in suspension after fibroblast attachment [39,40,41,42,43,44,45,46] and assessing their immunophenotype only subsequently [40,44,46]. In this context, our cell purification approach, which consists in the selection of TCs immediately after tissue processing and according to their immunophenotypic profile, may represent an innovative, reliable, and reproducible method to obtain a pure TC population suitable for in vitro analyses and other applications. 

Indeed, besides allowing an in-depth comprehension of their morphofunctional characteristics, effective protocols for the isolation of TCs appear even more essential, especially when considering that these stromal cells are increasingly gaining attention as possible candidates in the field of regenerative medicine [5,10,52,55]. Interestingly, tissue regenerative and therapeutic effects of TCs have already been demonstrated in a few in vivo studies reporting that intramyocardial transplantation of these cells in post-infarcted rats was able to decrease the infarct size and improve cardiac functions [69,70]. Finally, as recently proposed [71], the establishment of novel reliable methodologies for TC purification and in vitro expansion represents a prerequisite for the possible application of these cells in the setting up of three-dimensional model systems, such as engineered tissue/organ equivalents, which are emerging as innovative tools for translational research purposes.

In conclusion, in the present in vitro study, we established a simple and efficient method to obtain a pure population of human TCs that has the great potential to become an important tool for elucidating the biological effects and the possible research applications of these cells. Since, to develop this novel methodology, we employed human skin specimens, we are aware that further studies will be necessary to validate the efficiency and reproducibility of our immunomagnetic cell separation technique for the purification of TCs from different anatomical compartments. In addition, it should be pointed out that, in order to verify the effectiveness of our TC purification method, we herein analyzed cell morphology and molecular profile immediately after separation at first passages in culture. Therefore, in the perspective of employing isolated TCs in functional assays, it will be also necessary to assess if these cells are able to maintain their morphology and immunophenotype in long-term cultures.

## 4. Materials and Methods 

### 4.1. Skin Sample Collection

Skin samples were collected as waste material from plastic surgery of six healthy subjects at the Plastic and Reconstructive Microsurgery Center, Careggi University Hospital, Florence, Italy. The study was approved by the Comitato Etico Regionale per la Sperimentazione Clinica della Toscana—sezione AREA VASTA CENTRO, Florence, Italy (approval number: 16687_bio; approval date: 14 April 2020), and all subjects provided written informed consent. All steps were carried out under a biological hood and under sterile conditions. Skin samples, collected and transported in full growth medium composed of Dulbecco’s modified eagle medium (DMEM; catalog no. 11965084; Thermo Fisher Scientific, Waltham, MA, USA) supplemented with 10% fetal bovine serum (FBS), 100 U/mL of penicillin, and 100 U/mL streptomycin (catalog no. 15140122; Thermo Fisher Scientific, Waltham, MA, USA), were immediately processed or kept at 37 °C in a humidified CO_2_ incubator set to 5% CO_2_ for a maximum of 24 h until processing.

### 4.2. Skin Sample Processing and Cell Extraction 

Skin samples were removed from the medium and transferred, with the dermis facing upwards, in a sterile 100 mm Petri dish. After removing any remaining adipose tissue and damaged zones with a sterile scalpel, to reduce the risks of contamination, skin samples were retrieved with clean sterile tweezers and placed in a new sterile Petri dish with the epidermis on the top. Skin samples were, then, cut into small pieces of approximately 3 mm using a scalpel and successively covered and incubated in 7 mL of a dispase solution (5 U/mL; catalog no. 07913; Stemcell Technologies, Vancouver, Canada) overnight at 4 °C. After the incubation, skin samples were transferred into a new sterile Petri dish and, while maintaining the dermis in place with tweezers, the epidermis was delicately and slowly peeled off the dermis using thin curved tweezers and discarded. The remaining dermic pieces were collected in a 15 mL conical tube containing endothelial cell growth basal medium-2 (EBM-2; catalog no. CC-3156; Lonza, Basel, Switzerland) supplemented with 5% FBS and 0.015 g/mL collagenase (catalog no. C0130; Merck, Darmstadt, Germany) and incubated for 60 min at 37 °C in a thermostated water bath. After centrifuging at 300× *g* for 7 min to remove collagenase and placing the tissue pellet in a new Petri, a sterile slide was placed over the tissue pieces, slightly pushed, and overlaid with full growth medium. Cells were let to adhere to the Petri dish for a minimum of 24 h before slide removal and subsequently, subjected to magnetic-activated cell sorting (MACS) separation.

### 4.3. Two-Step Immunomagnetic Microbead-Based Cell Separation

Before proceeding to microbead-based cell separation, total cells were trypsinized, counted, centrifuged at 300× *g* for 7 min, and finally, resuspended to a maximum concentration of 1 × 10^7^ cells in 60 μL of starvation medium (EBM-2 basal medium supplemented with 2% FBS). For the isolation of the different cell populations (i.e., CD31+ ECs, CD31−/CD34+ TCs, and CD31−/CD34− fibroblasts), microbead-based cell separation was performed in two steps. The first step was carried out in order to isolate CD31+ cells (i.e., ECs), while the second step, performed on the remaining CD31− cells, allowed further separation of CD31−/CD34+ cells (i.e., TCs) from CD31−/CD34− cells (i.e., fibroblasts).

#### 4.3.1. CD31+ Endothelial Cell Isolation

CD31+ ECs were purified using the CD31 MicroBead Kit (catalog no. 130-091-935; Miltenyi Biotec, Bergisch Gladbach, Germany). Briefly, 20 μL of FcR Blocking reagent was added to total cell suspension and, after vortexing, cells were incubated with 20 μL of CD31 microbeads for 15 min at 4 °C. At the end of incubation, 1 mL of starvation medium was added to cell suspension, which was subsequently centrifuged at 300× *g* for 7 min, resuspended in 500 μL of starvation medium, and finally, subjected to magnetic separation. In particular, separation columns were placed in the magnetic field of a MACS Separator and washed with 3 mL of starvation medium before adding cell suspension. MACS columns contain a matrix composed of magnetic spheres covered with a cell-friendly coating. The space among the spheres is much larger than the size of the cells, allowing a free passage inside the column. When the column is placed in the MACS Separator, the strong magnetic field induced and amplified by the spheres within the column retains magnetic labeled cells, which do not bind directly to the column, but remain suspended within it, thus, undergoing reduced stress. Unlabeled cells (CD31− cells) were directly collected from the column effluent after three washes with 500 μL of starvation medium (negative selection), while magnetically labeled CD31+ cells were eluted from the columns by firmly pushing a plunger into the column outside the magnet (positive selection). CD31+ positive cells were cultured in EGM-2 MV complete medium (EGM-2 MV Microvascular Endothelial Cell Growth Medium-2 BulletKit; catalog no. CC-3202; Lonza, Basel, Switzerland), while CD31− cells were subjected to a second magnetic separation.

#### 4.3.2. CD31−/CD34+ Telocyte Isolation

CD31−/CD34+ TCs were isolated using the CD34 MicroBead Kit (catalog no. 130-046-702; Miltenyi Biotec, Bergisch Gladbach, Germany). Briefly, for positive selection, CD31− cells obtained from the first magnetic separation were centrifuged at 300× *g* for 7 min and resuspended in 300 μL of a buffer containing phosphate-buffered saline (PBS; pH 7.2), 0.5% FBS, and 2 mM ethylenediaminetetraacetic acid (EDTA). Cells were then incubated with 100 μL of FcR blocking buffer and 100 μL of CD34 microbeads for 30 min at 4 °C. Cells were washed by adding 5 mL of buffer and centrifuged at 300× *g* for 7 min. After aspirating the supernatant, cells were resuspended in 500 μL of buffer and subjected to the second magnetic separation. Unlabeled cells (CD31−/CD34− cells, i.e., fibroblasts) were directly collected from the column effluent after three washes with 500 μL of buffer (negative selection), while magnetically labeled cells (CD31−/CD34+ cells, i.e., TCs) were eluted from the columns by firmly pushing a plunger into the column outside the magnet (positive selection). TCs and fibroblasts were finally cultured in full growth medium composed of DMEM supplemented with 10% FBS, 2 mM l-glutamine (catalog no. BE17-605E/U1; Lonza, Basel, Switzerland), 100 U/mL of penicillin, and 100 U/mL streptomycin.

### 4.4. Cell Culture

CD31+ ECs, CD31−/CD34+ TCs, and CD31−/CD34− fibroblasts were cultured and maintained in 100 mm Petri dishes with the corresponding full growth medium until second passage. To assess cell morphology, after the first passage, phase-contrast images of the three cell populations were obtained under a Leica inverted microscope (Leica Microsystems, Mannheim, Germany) equipped with a digital camera.

### 4.5. Fluorescence Immunocytochemistry

ECs, TCs, and fibroblasts at the second passage in culture were seeded onto glass coverslips and fixed with 3.7% buffered paraformaldehyde. In order to confirm the efficacy of magnetic isolation of the three different cell populations, the expression of CD31 and CD34 markers was assessed by fluorescence immunocytochemistry. In particular, cells were permeabilized with 0.1% Triton X-100 in PBS, washed with PBS, and blocked with 1% bovine serum albumin in PBS for 1 h at room temperature. After blocking, cells were incubated overnight at 4 °C with mouse monoclonal anti-CD31 (catalog no. ab9498; Abcam, Cambridge, UK) and rabbit monoclonal anti-CD34 (catalog no. ab81289; Abcam, Cambridge, UK) antibodies, both at 1:50 dilution. The day after, slides were incubated in the dark for 45 min at room temperature with Rhodamine Red-X-conjugated anti-mouse and Alexa Fluor-488-conjugated anti-rabbit IgG (Invitrogen, Carlsbad, CA, USA) diluted 1:200. Double immunofluorescence staining was performed by mixing mouse anti-CD31 and rabbit anti-CD34 primary antibodies and subsequently mixing fluorochrome-conjugated secondary antibodies. Irrelevant isotype-matched and concentration-matched mouse and rabbit IgG (Sigma-Aldrich, St. Louis, MO, USA) were used as negative controls. Nuclei were counterstained with 4′,6-diamidino-2-phenylindole (DAPI). Immunolabeled cells were examined with a Leica DM4000 B microscope (Leica Microsystems, Mannheim, Germany) and fluorescence images were captured with a Leica DFC310 FX 1.4-megapixel digital color camera equipped with the Leica software application suite LAS V3.8 (Leica Microsystems, Mannheim, Germany). The same double fluorescence immunocytochemistry protocol was applied to assess the coexpression of CD34 and PDGFRα in isolated TCs by using rabbit anti-CD34 (1:50 dilution; catalog no. ab81289; Abcam, Cambridge, UK) in combination with mouse anti-PDGFRα (1:50 dilution; catalog no. ab35765; Abcam, Cambridge, UK). To estimate the purity of the immunomagnetically isolated CD31−/CD34+ TCs, the percentage of cells displaying both the CD31−/CD34+ immunophenotype and the characteristic TC morphology with respect to the total number of cells (i.e., cells with DAPI-labeled nuclei) was determined by cell counting in fifteen randomly chosen microscopic fields (20× original magnification) of each of the two CD31/CD34-immunostained slides per sample.

### 4.6. Wheat Germ Agglutinin Fluorescent Staining

To stain plasma membranes of cells at the second passage in culture, paraformaldehyde-fixed slides were rinsed in PBS and incubated for 10 min at room temperature in the dark with Alexa Fluor-488-conjugated WGA (catalog no. W11261; Thermo Fisher Scientific, Waltham, MA, USA) at 1:100 dilution. Nuclei were counterstained with DAPI.

### 4.7. Western Blotting

Whole cell protein lysates from the three cell populations at the second passage in culture were subjected to immunoblot analysis according to previously published protocols [72]. The following antibodies were used: Rabbit anti-CD31 (1:500 dilution; catalog no. ab28364; Abcam, Cambridge, UK), rabbit anti-CD34 (1:1000 dilution; catalog no. ab81289; Abcam, Cambridge, UK), mouse anti-PDGFRα (1:500 dilution; catalog no. ab35765; Abcam, Cambridge, UK), mouse anti-vimentin (1:1000 dilution; catalog no. M7020; Dako, Glostrup, Denmark), and mouse anti-glyceraldehyde 3-phosphate dehydrogenase (GAPDH; 1:5000 dilution; catalog no. ab8245; Abcam, Cambridge, UK). Immunodetection was performed using the Western Breeze Chromogenic Western Blot Immunodetection Kit (Invitrogen, Carlsbad, CA, USA) as described elsewhere [72].

## Abbreviations

TCs	Telocytes
TEM	Transmission electron microscopy
ECs	Endothelial cells
PDGFRα	Platelet-derived growth factor receptor α
WGA	Wheat germ agglutinin
GAPDH	Glyceraldehyde 3-phosphate dehydrogenase
DMEM	Dulbecco’s modified eagle medium
FBS	Fetal bovine serum
EBM-2	Endothelial cell growth basal medium-2
MACS	Magnetic-activated cell sorting
EGM-2	Endothelial cell growth medium-2
PBS	Phosphate-buffered saline
EDTA	Ethylenediaminetetraacetic acid
DAPI	4′,6-diamidino-2-phenylindole
